# Natural IgG Autoantibodies Are Abundant and Ubiquitous in Human Sera, and Their Number Is Influenced By Age, Gender, and Disease

**DOI:** 10.1371/journal.pone.0060726

**Published:** 2013-04-02

**Authors:** Eric P. Nagele, Min Han, Nimish K. Acharya, Cassandra DeMarshall, Mary C. Kosciuk, Robert G. Nagele

**Affiliations:** 1 Biomarker Discovery Center, New Jersey Institute for Successful Aging, University of Medicine and Dentistry of New Jersey, Stratford, New Jersey, United States of America; 2 University of Medicine and Dentistry of New Jersey-Graduate School of Biomedical Sciences at the School of Osteopathic Medicine, Stratford, New Jersey, United States of America; 3 Durin Technologies, Inc., New Brunswick, New Jersey, United States of America; Beth Israel Deaconess Medical Center, United States Of America

## Abstract

The presence of self-reactive IgG autoantibodies in human sera is largely thought to represent a breakdown in central tolerance and is typically regarded as a harbinger of autoimmune pathology. In the present study, immune-response profiling of human serum from 166 individuals via human protein microarrays demonstrates that IgG autoantibodies are abundant in all human serum, usually numbering in the thousands. These IgG autoantibodies bind to human antigens from organs and tissues all over the body and their serum diversity is strongly influenced by age, gender, and the presence of specific diseases. We also found that serum IgG autoantibody profiles are unique to an individual and remarkably stable over time. Similar profiles exist in rat and swine, suggesting conservation of this immunological feature among mammals. The number, diversity, and apparent evolutionary conservation of autoantibody profiles suggest that IgG autoantibodies have some important, as yet unrecognized, physiological function. We propose that IgG autoantibodies have evolved as an adaptive mechanism for debris-clearance, a function consistent with their apparent utility as diagnostic indicators of disease as already established for Alzheimer’s and Parkinson’s diseases.

## Introduction

Since the discovery of auto-reactive, natural antibodies more than two decades ago, a great deal of effort has been devoted to describing their generation, regulation, and function Much of this effort has focused on natural IgM antibodies. It has been determined that natural IgM antibodies are present in experimental animals completely deprived of potential antigen, and that their reactivity profiles are remarkably conserved between individuals [1]–[3]. These natural IgMs have also been shown to be universally present in human serum [4], [5]. The fact that some of these natural antibodies are auto-reactive has led to suspicions that they might function in maintaining tissue homeostasis [6], [7]. Subsequent investigations determined that IgM autoantibodies do indeed bind to common apoptotic neo-antigens, such as phosphatidylserine, cardiolipin, and annexin IV, and that they also recognize markers of cell senescence [8]–[12]. Given these patterns of reactivity, it is hypothesized that natural IgM antibodies serve as a conserved way to assist in the clean-up of apoptotic cellular debris.

Natural IgM antibodies are produced by the relatively class-restricted B-1 cells, while IgG antibodies are known to be produced via the T cell-dependent interactions of follicular B-2 cells [13]. The former are positively selected for when faced with self-antigen, while the latter is thought to be strictly held within the confines of self-tolerance [14]. Therefore, any presence of IgG autoantibodies in the blood is usually considered to be the result of a pathological breakdown in self-tolerance. This notion is supported by the fact that many autoimmune diseases, including rheumatoid arthritis, Sjögren’s syndrome, and systemic lupus erythematosis, are initiated or exacerbated by IgG autoantibodies to specific cellular and tissue components [15]-[17]. Thus, it is still generally assumed that all auto-reactive IgG are not purposeful products of the human immune system.

Although IgG autoantibodies in the blood can be a serological hallmark of autoimmune diseases, the low specificity of most of these disease-associations implies the presence of autoantibodies even in healthy individuals. An increasing number of studies have also revealed a link between autoantibodies and many non-autoimmune phenomena, like cancer and neurological disease [7], [15]–[18]. New serum autoantibodies are continuously being identified in the literature as related to various conditions, but most attempts to connect them more directly to known risk factors, pathogenesis, or prognosis are tenuous. This is often compounded by limited study methodologies and a singular focus on one individual disease process. A systemic investigation into the extent of natural serum IgG autoantibodies may help provide us with a clearer understanding of the role of these autoantibodies and their relationship with disease.

To accomplish this, we probed protein microarrays containing nearly 10,000 human proteins with sera collected from individuals of different ages, genders, and pathological states. All samples contained auto-reactive IgG, and the majority possessed autoantibodies to more than one thousand discrete human protein antigens. The total number detected was significantly influenced by gender, age, and the presence of specific diseases. Furthermore, the unique profile of autoantibodies present in an individual was relatively consistent over time. Rats and swine were also found to possess serum IgG autoantibodies and demonstrated similar stability in individual IgG autoantibody profiles over time. The number, diversity, and apparent conservation of IgG autoantibodies in mammals have led us to suggest that abundant IgG autoantibodies are a normal feature of the blood and, like auto-reactive IgM, they may play an important physiological role such as maintaining tissue homeostasis through adaptive debris-clearance as suggested by Avrameas nearly two decades ago [6]. Indirect evidence in support of this concept comes from our recent studies showing that small panels of disease-specific autoantibodies, presumably linked to debris clearance, can be used for the detection and diagnosis of diseases such as Alzheimer’s and Parkinson’s diseases [19], [20].

## Materials and Methods

### Human Serum Samples

Healthy control sera were purchased from the following vendors: *Analytical Biological Systems, Inc*. (Wilmington, DE), *Bioserve* (Beltsville, MD) and *Asterand, Inc*. (Detroit, MI). Parkinson’s disease (PD) and Alzheimer’s disease (AD) serum samples were obtained from *Analytical Biological Systems, Inc*. (Wilmington, DE), *ProteoGenex* (Culver City, CA) and *PrecisionMed* (Solana Beach, CA). Breast cancer (BC) and multiple sclerosis (MS) serum samples were obtained from *Asterand, Inc*. All samples were handled by standard procedures and stored at -80°C. Demographic characteristics of the study population are shown in Tables 1 and 2. Approval for the use of blood samples for this study was obtained from the UMDNJ-Stratford Institutional Review Board.

**Table 1:** Effects of Age and Gender on Autoantibody Count.

Age	N	% Female	Antibody Count	Comparison: P value
< 45	10	33.3	1498.2 ± 545.7	<45 vs. 45–65: 0.0021
45 – 65	32	18.2	2335.6 ± 1009.5	45–65 vs. >65: 0.37
> 65	15	60	2647.8 ± 1139.2	<45 vs. >65: 0.0028
**Sex**	**N**	**Age**	**Antibody Count**	**P value**
Female	18	57.6 ± 18.7	2772.5 ± 714.8	0.004
Male	39	53.1 ± 15.1	2039.3 ± 1092.7	

A total of 57 normal healthy controls were split into three age groups and two gender groups. The percentage of female subjects in each group is indicated. A Z-factor of greater than 0.4 was used to define a positive antibody response. The mean and standard deviation of antibody counts are shown. The difference of the antibody counts between indicated groups were tested by student t test and p value as shown.

**Table 2:** Effects of Health Status on Autoantibody Count.

	N	Age	% Female	Antibody Count	P value
**AD**	36	79.4 ± 9.1	58.3	1515.2 ± 695.5	7.64E-06
**Control**	47	61.1 ± 8.2	31.9	2435.3 ± 1050.5	
**PD**	48	68.0 ± 9.7	35.4	1833.4 ± 900.4	3.50E-03
**Control**	47	61.1 ± 8.2	31.9	2435.3 ± 1050.4	
**Breast Cancer**	18	47.4 ± 5.5	100	2491.6 ± 1589.1	0.71
**Control**	15	53.8 ± 18.1	100	2645.9 ± 703.3	
**Multiple Sclerosis**	7	48.4 ± 8.6	71.4	2093.1 ± 8.6	0.044
**Control**	10	52.5 ± 4.0	50	3118.9 ± 884.4	

Several diseased groups (Alzheimer’s disease, Parkinson’s disease, breast cancer and multiple sclerosis) and the corresponding age-and-gender matched controls were studied. The age (mean ± standard deviation), percentage of female subjects, number of positive autoantibodies (mean ± standard deviation) are shown for each group. The difference of the autoantibody counts between diseased and corresponding control groups were tested by student t test with p value shown.

### Human Protein Microarrays

To assess the complexity of autoantibody profiles in human serum, we used human protein microarrays (Cat. *No*. PAH0525020, *Invitrogen*, Carlsbad, CA, USA), each containing 9,486 full-length human protein antigens (www.invitrogen.com/protoarray). All proteins have been expressed as GST fusion proteins in insect cells, purified under native conditions, and spotted in duplicate onto nitrocellulose-coated glass slides. All arrays were probed and scanned according to the manufacturer’s instructions using commercially prepared reagents. Briefly, microarray slides were blocked (Blocking Buffer, Cat. *No. PA055*, *Invitrogen*) and then incubated with serum samples diluted 1∶500 in washing buffer. After washing, the arrays were probed with anti-human IgG (H+L) conjugated to AlexaFluor 647 (Cat. *No*. A-21445, *Invitrogen*). Arrays were then washed, dried, and immediately scanned with a GenePix 4000B Fluorescence Scanner (*Molecular Devices*, Sunnyvale, CA, USA).

### Protein Microarray Data Analysis

The fluorescence data for each microarray was acquired by *Genepix Pro* analysis software after scanning. The resulting *Genepix Results* (GPR) files were then imported into Invitrogen’s *Prospector 5.2* for analysis. Background subtraction and outlier detection were all performed by the ‘compare’ function of *Prospector*. The Z-Factor for a pair of protein features indicates how far the mean of that feature pair deviates from the mean of the negative controls in each sub-array. A Z-factor of greater than 0.4 was used to define a positive signal. Redundant BSA spots within each sub-array served as negative controls, with a required signal of less than 1000 RFUs to be considered valid for this study. All data is MIAME compliant and the raw data has been deposited in *Gene Expression Omnibus* (GEO) under acquisition number GSE39087 (http://www.ncbi.nlm.nih.gov/geo/). Cross-group comparison of the number of autoantibodies was done using the Student’s t-test.

### Dot Blot Analysis

Purified recombinant human ICAM4 (0.134 µg/µl) and PTCD2 (0.098 µg/µl) proteins (Cat. No. TP300636 and TP315253, *OriGene Technologies*, Inc., Rockville, MD), were spotted onto a nitrocellulose membrane in 1 µl volumes. The proteins were blocked in 5% non-fat milk for one hour at room temperature (RT) and then probed with serially diluted (1∶500, 1∶1000 and 1∶2000) human serum samples for one hour at RT. All sera were identical to those used on the human protein microarrays. The dot blots were then reacted with anti-human IgG (H+L) HRP conjugate antibody (Cat. No. 31410, *Thermo Fisher Scientific Inc*., Pittsburgh, PA, USA) for one hour at RT, incubated with ECL reagent (Cat. No. 34096, *Thermo Fisher Scientific Inc*., Pittsburgh, PA) for one minute, and then exposed to autoradiography film.

### Western Blot Analysis

Western blot analysis was performed to determine the number, diversity, stability over time and targets of serum autoantibodies using methods previously described [21]. Fresh frozen rat brain, pig brain, and frozen human brain tissue were lysed using RIPA buffer (150 mM sodium chloride, 1.0% Triton) or 2% Triton X-100 buffer. Normal human liver, lung, and kidney tissue lysates were purchased from *PROTEIN Inc*. (Cat. No. HN-13, HN-15, HN-11, Romona, CA). All sera were diluted 1∶500 and used as primary antibodies individually. Secondary antibodies were HRP-conjugated anti-human IgG (H+L) (Cat. No. 31410, *Thermo Fisher Scientific Inc*., Pittsburgh, PA), anti-rat IgG and anti-pig IgG (Cat. No. STAR21B and AAI41P, AbD *SeroTec*, Oxford, UK). Western blots were then developed using ECL reagent and autoradiography film. Approval for the use of all animal samples in this study was obtained from the UMDNJ-Stratford Institutional Animal Care and Use Committee.

## Results

### IgG Autoantibodies are Abundant and Ubiquitous in Human Sera

**Figure 1:**
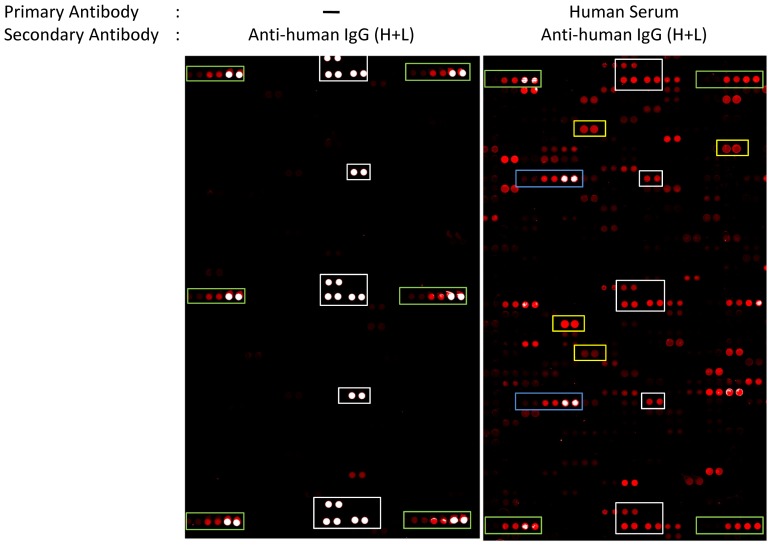
Representative images of human protein microarrays. (Left) Small portion of a protein microarray containing 9,480 native human proteins probed with blocking buffer and secondary antibody as a quality control. (Right) Protein microarray probed with an individual human serum sample under the same conditions. Manufacturer’s controls (white boxes), gradient of IgG positive controls (green boxes), anti-human IgG controls (blue boxes) and examples of immunopositive reactions (yellow boxes) are indicated. In this study, all arrays were scanned at PMT setting of 600. Under this condition, the relative fluorescence unit (RFU) at saturation is around 75000 as exemplified by IgG controls (green boxes). The RFUs of BSA negative controls are under 1000. The coefficient of variance between duplicate spots averaged 4.7% ∼ 8.8%.

**Figure 2:**
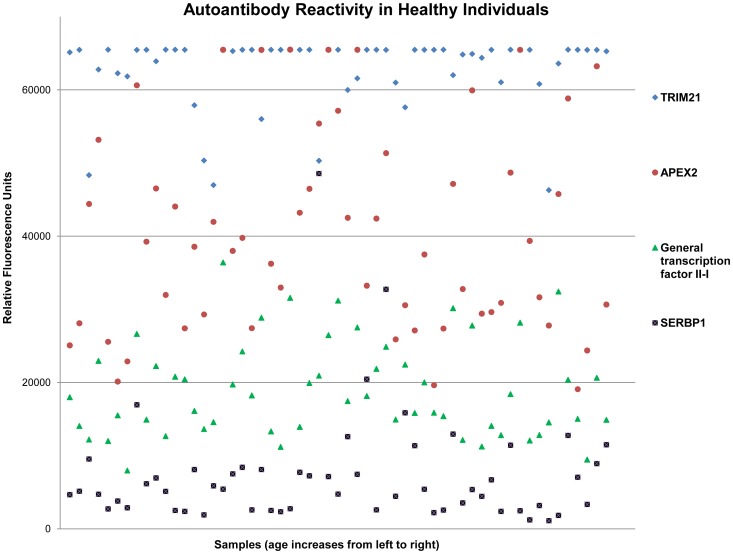
Representative autoantibody data showing their relative abundance in healthy individuals. An example of the reactions of four highly prevalent autoantibodies in a population of healthy subjects is presented. The X axis indicates individual samples arranged in order of increasing age from left to right. Relative fluorescence units (RFUs) (Y axis) reflect the resulting antigen-antibody reaction detected on the protein microarrays. Four specific autoantigens [tripartite motif-containing 21 (TRIM21), APEX nuclease (apurinic/apyrimidinic endonuclease) 2 (APEX2), General transcription factor II-I and SERPINE1 mRNA binding protein 1 (SERBP1)] were chosen due to their high prevalence in > 60% of the healthy population. The clear lamination of signal intensities shown by each autoantibody-antigen reaction suggests comparable levels of these autoantibodies in the blood across the population

To detect and determine the relative abundance of autoantibodies in the blood, we probed human protein microarrays with individual human sera from the following subject groups: normal healthy controls (n = 57), Alzheimer’s disease patients (n = 36), Parkinson’s disease patients (n = 48), multiple sclerosis patients (n = 7), and breast cancer patients (n = 18) for a total of 166 human serum samples. Each microarray contained 9,486 native human proteins bound to a nitrocellulose substratum as potential targets for serum IgG (Fig. 1). A Z-factor score of ≥ 0.4 in the resulting relative immunofluorescence indicated a mean of 1,996.9 different IgG-bound antigens per individual; although with significant individual variation (n = 166, 1,996.9 ± 1,051.9). The specific antigens and the relative abundance of their corresponding autoantibodies are shown in Figure 2 as well as in Table S1. To further focus on the common and abundant autoantibodies across the population, we set the analytical criteria as follows: (1) Z factor ≥ 0.4; (2) RFU > 5000; and (3) consistently positive in over 60% of subjects in each group, including control and diseased groups. Interestingly, using these criteria, we found 66 autoantibodies that are common and abundant in the population, regardless of age, gender and disease status (Table S1). These autoantibody targets are involved in various biological functions including metabolism, signal transduction and structural proteins. No individual tested had less than 301 distinct serum autoantibodies detectable using these criteria: most had thousands.

**Figure 3:**
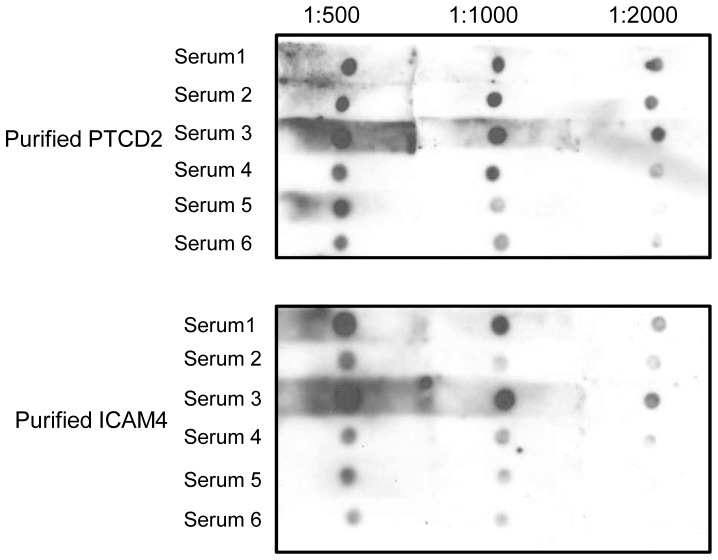
Dot blot confirmation of serum IgG auto-reactivity. Two identified antigens of serum autoantibodies, PTCD2 and ICAM4, were spotted onto nitrocellulose and probed with serum identical to that used on the protein microarrays. Serial dilution with diminishing immunoreactivity confirmed the presence and activity of IgG autoantibodies in human sera.

To verify the reactivity of autoantibodies detected with human protein microarrays, we carried out dot-blot analyses using commercially-obtained purified, native proteins. We selected the antigens of two commonly detected autoantibodies, pentatricopeptide repeat-containing protein 2 (PTCD2) and intercellular adhesion molecule 4 (ICAM4), and sought to confirm autoantibody reactivity. Each protein was spotted onto a nitrocellulose membrane in equal amounts and then probed with sera identical to that used to probe the microarrays (Fig. 3). Results show reactivity and proportionately diminished reactions with serial dilutions, thus providing additional biochemical confirmation that the autoantibodies detected in human sera are present and reactive to their antigens.

### Age, Gender, and the Presence of Specific Diseases Strongly Influence the Number of Autoantibodies Detected Using Protein Microarrays

In an effort to represent the broadest possible population, the serum samples used to probe human protein microarrays included subjects of each gender from a variety of ages and health backgrounds. Data showing the effects of these variables on the total number of IgG autoantibodies detectable using human protein microarrays are presented in Tables 1 and 2.

To investigate the influence of age on the average number of serum autoantibodies detected, healthy control samples of each gender were categorized into three groups: <45 years, 45–65 years, and >65 years (Table 1). Results revealed that increasing age was accompanied by a corresponding increase in the total number of detectable autoantibodies. The >65 year old group had the highest number of detectable autoantibodies (2647.8 ± 1139.2, n = 15); the 45–65 years group had the next highest number of serum autoantibodies (2335.6 ± 1009.5, n = 32); and the youngest age group, <45 years, had the fewest autoantibodies (1498.2 ± 545.7, n = 10). The lesser number of autoantibodies in the <45 years group was statistically significant when compared to both the 45–65 years group (p = 0.0021) and the >65 years group (p = 0.0028). Taken together, this indicates that there is a somewhat linear progression of increasing serum autoantibody diversity with increasing age.

When examined on the basis of gender, healthy age-matched samples from males and females exhibited a striking difference in the average number of serum autoantibodies (Table 1). Females were found to have a substantially greater number of autoantibodies (2772.5 ± 714.8, n = 18) than males of equivalent age and health status (2039.3 ± 1092.7, n = 39). This difference was statistically significant (p = 0.004) and may, at least in part, account for the increased prevalence of autoimmune diseases in women [22].

The presence of specific diseases also strongly influenced the number of detectable autoantibodies in the serum. Data for each disease subgroup examined compared to gender- and age-matched controls are shown in Table 2. Alzheimer’s and Parkinson’s disease patients demonstrated a statistically significant reduction in the number of autoantibodies compared to healthy controls (p = 6.0×10^−5^ and p = 0.023, respectively). Each, on average, had several hundred fewer autoantibody-bound antigens represented on protein microarrays than their healthy counterparts. Multiple sclerosis patients had a significant reduction in the average number of autoantibodies as well (p = 0.044). Lastly, while breast cancer patients had fewer detectable autoantibodies than their matched controls, the difference was not significant (p = 0.71).

### Serum Autoantibodies are Reactive to Proteins Expressed in Many Human Tissues

**Figure 4:**
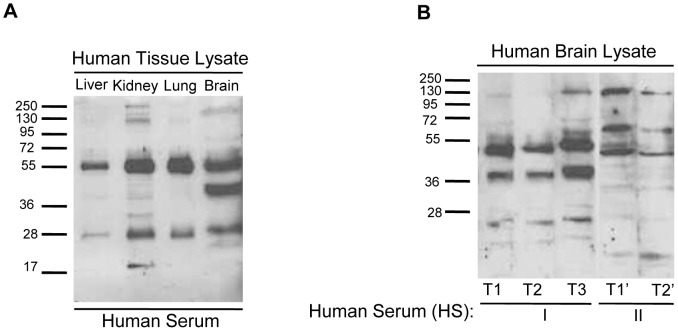
Serum IgG autoantibodies consistently bind to diverse antigens in many human organs. A. Human liver, kidney, lung, and brain tissue lysates were separated by SDS-PAGE and then probed with human serum. As indicated by the many immunoreactive protein bands of differing molecular weights, serum IgG autoantibodies bind to a variety of common and organ-specific SDS-denatured antigens. **B**. In western blots, proteins from human brain extract were probed with human serum (HS-I and HS-II) as primary antibodies. Serum samples at three timepoints, T1, T2 and T3, were collected from the same individual (HS-I) over a period of four years. Serum samples T1’ and T2’ were collected from a different individual (HS-II) over a period of two years. Consistent patterns of serum IgG immunoreactivity were specific to individuals and appeared to remain stable over time.

As described above, human protein microarrays revealed that human serum contains IgG autoantibodies directed against thousands of distinct protein antigens. These proteins are expressed throughout the body and are involved in many critical processes. We next sought to confirm that the autoantibodies detected are reactive to antigens in diverse human tissues and organs using western analysis (Fig. 4). To investigate the reactivity of serum autoantibodies to tissue-specific epitopes, proteins from human liver, kidney, lung, and brain tissue lysates were separated by SDS-PAGE and probed with individual human serum samples as primary antibody (Fig. 4A). All sera tested were found to possess autoantibodies that were immunoreactive to multiple proteins from each of the organs. Not surprisingly, many autoantibodies bound to common antigens shared by each organ as well.

### Autoantibodies are Abundant in the Blood of All Mammalian Species Tested

**Figure 5:**
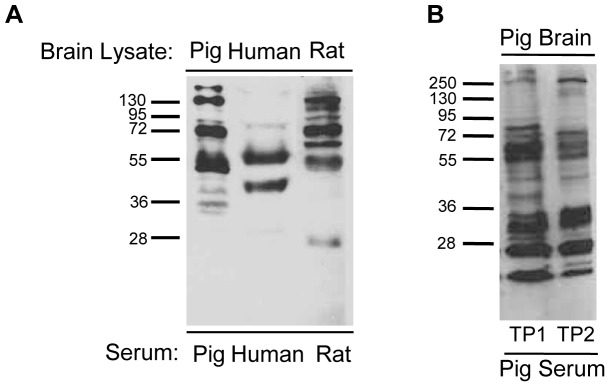
Serum IgG autoantibodies are detected in all mammals tested. **A**. Pig, human, and rat brain lysates were separated by SDS-PAGE and then probed with their respective species-specific serum as a primary antibody. Each species possessed IgG autoantibodies that bound to a variety of SDS-denatured brain self-antigens. **B**. Proteins from pig brain extract were probed with serum collected from a single pig at two timepoints, TP1 and TP2, spanning three months. The majority of the pig auto-reactive IgG antibodies remain the same.

To investigate whether autoantibodies with affinity to diverse self-antigens are also abundant and ubiquitous in the sera of other mammals, we separated proteins from rat and pig brain lysates via SDS-PAGE and probed them with their respective sera as primary antibodies (Fig. 5A). Both rat and pig serum were also found to possess numerous autoantibodies that were self-reactive to their own brain antigens. Interestingly, in western blots, human sera demonstrated comparatively less immunoreactivity to human brain antigens than did the corresponding blots using rat and pig lysates and sera. This reduction is most likely due to unavoidable post-mortem protein degradation which can be avoided in the animal models. The intensity and comparable number of immunopositive reactions on western blots using rat and pig serum lead us to conclude that, like humans, both animals have abundant serum IgG autoantibodies and that perhaps complex serum IgG autoantibody profiles are a conserved feature among mammals.

### Individual Autoantibody Profiles Remain Consistent Over Time

We next sought to investigate the stability of individual autoantibody profiles over time. To address this, proteins from human brain tissue lysate were separated by gel electrophoresis and probed with human serum obtained from individuals at several time points over a period of years (Fig. 4B). Similarly, the stability of autoantibody profiles over time was also tested in pigs over a period of three months (Fig. 5B). The first human subject (HS-I) was a fifty-two year old male followed over a period of four years. The second (HS-II) was a sixty-two year old female with Parkinson’s disease followed for two years. As shown in Figure 4B, although there are slight variations in individual band intensity from one blood withdrawal date to the next, the overall autoantibody reactivity pattern, reflecting the presence of specific autoantibodies, remained remarkably stable over the entire period. These findings lead us to conclude that individual IgG autoantibody profiles in the blood are stable over long periods of time, which is consistent with the earlier findings of Lacroix-Desmazes et al. [23].

## Discussion

Originally thought to bind exclusively to foreign antigens, we now understand that there is a spectrum of auto-reactivity in serum antibodies: from the innately produced, poly-reactive IgMs that function to clear the tissues of post-apoptotic debris, to the pathological IgGs that are associated with autoimmune diseases. The former represent “natural” antibodies produced by a unique population of B-1 cells from birth in an apparent default pattern without the need for antigen presentation [1]–[4]. IgG autoantibodies, however, are generally thought to represent a failure in B cell self-tolerance mechanisms or an extremity of somatic hypermutation, and are generally not considered a purposeful product of the healthy immune system [24]. Despite this, it has been suggested that IgG autoantibodies are both abundant and ubiquitous in human sera, and that some may be present in the absence of disease and may arise from an exaggerated immune response against self as seen in autoimmune diseases [7], [18]. Here, we demonstrate that all human sera possess extremely complex profile of IgG autoantibodies directed against thousands of discrete human protein antigens. This level of complexity alone suggests that IgG autoantibodies carry out some important, but as yet unrecognized, function.

### IgG Autoantibody Abundance

An accurate estimate of the total number of autoantibodies in human serum at any one time is a difficult undertaking. The development of multiplex platforms containing a large number of human protein antigens such as protein microarrays, multiplex ELISA, and luminex beads now make this task possible. We chose human protein microarrays because they currently contain the largest number of antigens in a native conformation: 9,486 distinct human antigens. Using a standard Z-factor cutoff of ≥ 0.4, relative immunofluorescence values indicated that most individuals have thousands of circulating serum IgG autoantibodies (Tables 1, 2). Considering that the 9,486 proteins on the microarray represent only a fraction of the estimated size of the entire human proteome, it is logical to assume that there are actually far more autoantibodies present in serum than detected here, perhaps numbering in the many thousands. Furthermore, this analysis considered autoantibody diversity only from the perspective of unique antigens bound: the polyclonal nature of human antibodies makes a more precise estimate of distinct idiotypes difficult. However, these and other technical limitations would all tend to reduce the total number of detectable autoantibodies. Given the strong evidence supporting an abundance of autoantibodies in all human sera, it is probable that there are many more naturally occurring autoantibodies than detected even here: all with cognate antigens representing a tremendous variety of human proteins.

### The Influence of Age, Gender, and Disease on IgG Autoantibody Diversity

Previous studies using quantitative immunoblotting have shown that the self-reactive IgM autoantibody repertoire differentiates during the first years of life and remains relatively constant thereafter [25]. There is evidence that this combinatorial immune recognition repertoire is present in all mammals and that its origin may date back to an early point in the evolution of jawed vertebrates as a rapid genetic process that is independent of antigenic selection [26]. Here, we show that while all humans also have a great number of serum IgG autoantibodies, this number can vary considerably between individuals. We have investigated the influence of three factors, age, gender and disease, on the number of detectable autoantibodies using protein microarrays. Surprisingly, the number of IgG autoantibodies present in the serum increases almost linearly with age (Table 1). This is a significant observation: it contrasts directly with serum diversity of auto-reactive natural IgM which, along with its progenitor B-1 cell populations, is thought to gradually diminish with age [27]. Gender was also found to be an important variable. Females have a much higher average number of autoantibodies than males (p = 0.004) (Table 1). This finding is relevant in light of the fact that females suffer from autoimmune misregulation at much higher rates than men [22]. Lastly, disease itself seems to exert an effect on the number of autoantibodies in serum. Alzheimer’s disease, Parkinson’s disease, and multiple sclerosis patients all had a statistically significant decrease in the number of autoantibodies when compared to age- and gender-matched controls (Table 2). Even breast cancer patients had a measurable decrease, although it was less significant. The diminished autoantibody counts among the Alzheimer’s and Parkinson’s disease subgroups were particularly striking, given that those patients were older and thus would have otherwise fallen into the demographic group with the highest number of autoantibodies. Further research is needed for nuanced understanding of the influence of age, gender, and health-status in autoimmunity.

### Autoantibody Abundance: A Physiological Necessity?

Here, we show that all human sera possess thousands of IgG autoantibodies regardless of age, gender, or the presence of disease. We have also confirmed that complex profiles of auto-reactive IgGs are consistent in individuals over long time periods as shown previously by Lacroix-Desmazes et al. [23] and Mirilas et al. [28] and are conserved in other mammalian species. Further research is needed to understand exactly how these autoantibodies are produced and sustained. It is probable that some of them might have an infectious origin; being rendered self-reactive through the vagaries of somatic hypermutation. But, given the vast metabolic expense of maintaining the clonal B cell populations necessary to support such a stable and complex profile of thousands of autoantibodies, it is unlikely that all of these autoantibodies represent a pathological breakdown in central tolerance. Rather, we suggest that this diversity and consistency indicate that IgG autoantibodies are necessary and perform some key physiological function.

The supposition that autoantibodies may actually perform some beneficial function is not without precedent. As mentioned above, the debris-clearing activity of IgM autoantibodies has been well established, and mice experimentally deprived of these autoantibodies demonstrate lupus-like symptoms [29], [30]. Even auto-reactive IgGs have received some attention in this regard [7]. Vargas and colleagues have already demonstrated the importance self-reactive IgG antibodies in the central nervous system. Anti-myelin IgG autoantibodies assist in debris-clearance after peripheral nerve injury, and, in their absence, axonal regeneration is impeded [31]. Another study on the plasma IgG and IgM levels in children with autism spectrum disorders seems to indicate that immunoglobulins are involved in the development of a healthy brain. Children with autism have significantly reduced levels IgG and IgM, and this reduction correlates with the severity of behavioral deficit [32]. This evidence, combined with the vast numbers of IgG autoantibodies measured in even healthy human serum blurs what was once thought to be a linear relationship between auto-reactive IgG and autoimmunity. There does seem to be a role for IgG autoantibodies in the healthy immune system.

### IgG Autoantibodies May Represent an Adaptive Response to Injury and Disease

It is important to differentiate the potential homeostatic functions of auto-reactive IgG from those of natural IgM. Considering the complexity of follicular B-2 cell activation, and the adaptive nature of the antibody response, IgG autoantibodies would be perfectly suited to clear the debris from situation-specific events like disease or trauma. On the other hand, the limited, pre-programmed panel of innate natural IgM would be incapable of this kind of tailored response.

Avrameas and coworkers have suggested that the recognition of self-constituents by the immune system may play a role in body homeostasis [6], [7]. In accord with this concept, we extend this hypothesis by proposing that IgG autoantibodies function as an adaptive mechanism to clear intercellular debris, thus implying that disease-induced tissue damage leads to increased production of autoantibodies appropriate to the clean-up of that debris. Thus, under conditions of ongoing pathology, autoantibodies that target the unique pathology-specific debris should show selective and measureable titer increases in the blood [33]–[35]. In agreement with this concept, the results of our previous studies have demonstrated that it is possible to identify and make use of disease-induced changes in autoantibody profiles as diagnostic biomarkers of disease. For example, we have recently shown that separate panels of autoantibody biomarkers can be used to successfully and accurately identify and diagnose Alzheimer’s and Parkinson’s disease patients from controls with high degrees of sensitivity and specificity [19], [20]. We propose that, in these diagnostics, we are measuring specific changes in autoantibody profiles that are associated with the autoantibody response to the very specific damage induced by these particular pathologies. Since many diseases exhibit cell- and tissue-specific damage, we predict that the identification of characteristic disease-induced changes to autoantibody profiles can be used as successful diagnostics for a wide variety of diseases.

## Conclusion

IgG autoantibodies are abundant and ubiquitous in the serum of all humans. Further evidence indicates that this complex profile of autoantibodies is present in other mammals and consistent in individuals over time. We suggest that the scale and universality of autoantibody proliferation in all sera is a telltale sign of its necessity – perhaps as an adaptive debris-clearance mechanism. Moreover, we propose that disease-induced perturbations in individual autoantibody profiles present an opportunity to accurately detect and successfully diagnose disease, as we have already demonstrated with Alzheimer’s and Parkinson’s diseases. Lastly, we suggest that the widespread presence of these naturally occurring, non-pathological IgG autoantibodies presents a new frontier in immunological research. It prompts future exploration of the potential of these autoantibodies as a new class of biomarkers for diagnostics and therapeutics.

## Supporting Information

Table S1 Positive autoantibody lists determined by different selection criteria.
(XLSX)
